# Supplementary material for: Vitamin D Modified DSS-Induced Colitis in Mice via STING Signaling Pathway
Source: Biology (Basel). 2025 Jun 18;14(6):715. doi: 10.3390/biology14060715 (PMC12190092; doi:10.3390/biology14060715)
Supplement: Supplementary file 1 [file biology-14-00715-s001.zip › Supplementary Materials S1: 16s-report of gut microbiota/Groups/diff_analysis/UnivarTestFamily/figure/barplot/alllog.pdf]

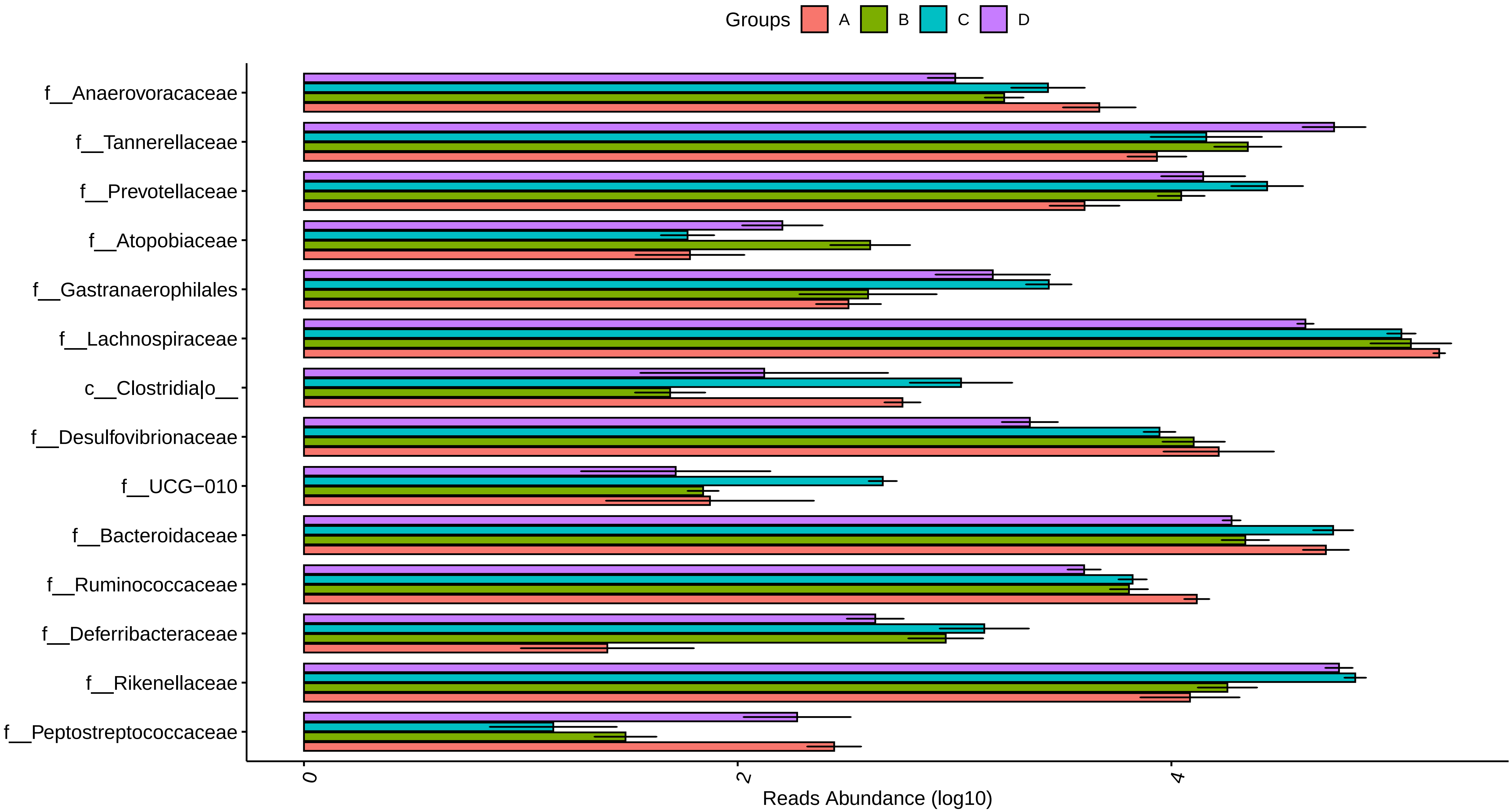

Groups
A
B
C
D
f__Anaerovoracaceae
f__Tannerellaceae
f__Prevotellaceae
f__Atopobiaceae
f__Gastranaerophilales
f__Lachnospiraceae
c__Clostridia|o__
f__Desulfovibrionaceae
f__UCG−010
f__Bacteroidaceae
f__Ruminococcaceae
f__Deferribacteraceae
f__Rikenellaceae
f__Peptostreptococcaceae
0
2
4
Reads Abundance (log10)
